# Antibiofilm efficacy of phage W5 against antimicrobial-resistant *Salmonella* Typhimurium targeting biofilms in dairy/meat/egg and on food-processing surfaces (PP/PE)

**DOI:** 10.1128/aem.01878-25

**Published:** 2026-03-26

**Authors:** Qian Chong, Qing Cao, Xi Wang, Ziqiu Fan, Yonghui Ma, Kunzhong Zhang, Jing Deng, Xuehui Zhao, Ji Zhi, Haohao Zhang, Kaihui Yang, Huiwen Xue, Huitian Gou

**Affiliations:** 1 College of Veterinary Medicine, Gansu Agricultural University 739715, Lanzhou, Gansu, China; Anses, Maisons-Alfort Laboratory for Food Safety, Maisons-Alfort, France

**Keywords:** phage, *Salmonella *Typhimurium, PP/PE, biofilm

## Abstract

Lytic phage W5, a novel isolate derived from poultry/livestock slaughterhouse effluent, displayed potent lysis against nine *Salmonella* serovars (e.g., *Salmonella* Typhimurium [*S*. Typhimurium], *Salmonella* Choleraesuis, and *Salmonella* Typhi). Integrated morphometry and genomic phylogeny affirmed its taxonomic placement within *Cornellvirus* (*Guernseyvirinae* and *Myoviridae*). Biological characterization demonstrated that under an optimal multiplicity of infection (MOI) of 1, the latent period was only 22 min, with the phage titer peaking at 3.67 × 10¹¹ PFU/mL during the logarithmic lysis phase (22–70 min). The phage retained stability at 50°C and across an extreme pH range (3–13) while achieving 98% host adsorption within 13 min in SM buffer/glycerol. In food applications, crystal violet staining confirmed that W5 significantly inhibited the growth of *S*. Typhimurium CMCC 50115 in milk, pork, and fresh eggs (eggshells and egg liquid), effectively preventing biofilm formation and eradicating preformed biofilms. Confocal laser scanning microscopy (CLSM) further verified efficient clearance of both nascent and mature biofilms formed by *S*. Typhimurium CMCC 50115 after phage W5 treatment under varied temperatures. Field emission scanning electron microscopy (FESEM) revealed potent lytic efficacy of the phage against biofilms on food-contact surfaces such as polypropylene (PP) and polyethylene (PE), with efficacy modulated by biofilm developmental stage and temperature, most prominently demonstrating superior eradication of early-stage biofilms on PE surfaces at 30°C. These findings collectively highlight phage W5’s substantial potential as a novel biocontrol agent against *Salmonella* in the food industry, attributable to its broad-spectrum lytic potency, exceptional environmental resilience, and multi-mechanistic biofilm-clearing properties.

**IMPORTANCE:**

Antimicrobial-resistant *Salmonella* poses a severe threat to food safety and public health, particularly through persistent biofilms on food-processing surfaces and animal-derived products. Traditional disinfection methods often fail against biofilms, while antibiotic overuse accelerates resistance. Bacteriophages offer a targeted, sustainable alternative, but their real-world efficacy in food systems remains underexplored. This study addresses critical gaps by evaluating a novel phage's potential to combat biofilms in relevant food matrices (dairy, meat, and eggs) and on common food-contact surfaces (PP/PE). By demonstrating practical biocontrol under varying storage conditions, this research advances phage-based strategies to enhance food safety while mitigating antimicrobial resistance—a priority for global health and sustainable agriculture. The findings have direct implications for industry sanitation and regulatory approvals for phage applications in food systems.

## INTRODUCTION

*Salmonella*, a globally significant foodborne pathogen, ranks third among fatal diarrheal diseases in humans and causes major economic losses in poultry (1). Widespread in livestock and wildlife, it primarily transmits via contaminated animal-derived foods like poultry, eggs, and dairy, causing acute fever, abdominal pain, diarrhea, and nausea. Poultry, especially highly susceptible young chicks, is a primary source of human infection, hindering intestinal health and poultry development. Key serotypes with global prevalence are *S*. Typhimurium and *Salmonella* Enteritidis (2). These pathogens cause an extremely high disease burden; in the US alone, annual estimates exceed 93 million related cases, 155,000 deaths, and 40,000 salmonellosis patients (3). According to 2023 CDC FoodNet surveillance data (covering 16% of the U.S. population, approximately 53.6 million people), a total of 8,454 *Salmonella* infection cases were reported. Laboratory testing successfully isolated bacterial strains in 83% of cases (7,015 cases), with 89% (6,243 cases) of these isolates undergoing complete serotyping. Clinical data showed a hospitalization rate of 29.1% (2,456 cases) and a case fatality rate of 0.65% (55 cases) (4). The foremost current challenge is antibiotic overuse in poultry farming. As evidenced by the surveillance of clinical isolates, this overuse accelerates the development of antibiotic resistance and the emergence of multidrug-resistant strains, posing a severe public health threat. Consequently, novel resistance mechanisms, such as β-lactamases and MCR (mobile colistin resistance) enzymes (5), have been continuously discovered and thoroughly investigated. Guo’s research on non-mobile colistin resistance determinants (NMCRs) has further deepened our understanding of colistin resistance through the characterization of novel NMCRs, such as NMCR-3, NMCR-4, and NMCR-5. For the first time, it elucidates the evolutionary progenitors of the MCR family and transforms our comprehension of colistin resistance mechanisms (6).

Microorganisms typically exist in aggregate states, with cells within their communities undergoing transitions from planktonic states to aggregate formation, ultimately forming biofilms. In the food industry, mixed bacterial biofilms pose particularly severe hazards. They enhance bacterial adhesion to food contact surfaces and exhibit high resistance to conventional disinfectants, leading to significant economic losses and health risks (7, 8). This resistance primarily stems from altered metabolic activity within the biofilm, the expulsion of antimicrobial agents via efflux pumps, and changes in resistance genes. These biofilms not only persist in liquid environments but also firmly adhere to food contact surfaces (9), posing a persistent challenge to food safety control. Consequently, inhibiting biofilm formation and eliminating established biofilms represent primary objectives for controlling biofilm hazards in the food industry (10).

The persistent escalation of *Salmonella* antimicrobial resistance constitutes a major global public health threat, where epidemiological surveillance data from England and Wales during 1996–2000 reveal explosive growth in ciprofloxacin resistance rates among non-typhoidal *Salmonella* serovars, specifically documenting peaks of 8% in *Enteritidis,* 39% in *Virchow,* and 70% in *Hadar*. Fluoroquinolone antibiotics in poultry and cattle farming have been shown to enhance the spread of drug-resistant strains. Phage typing further reveals the association between antibiotic resistance and the transfer of specific genes (11). Recently, Akshay and colleagues discovered that *Salmonella* develops multidrug resistance through synergistic efflux pump-porin interactions combined with target site mutations, necessitating comprehensive elucidation of these sophisticated drug evasion mechanisms for developing structurally robust novel antimicrobial molecules (12); concurrently, the 2024 European Foodborne Pathogen Surveillance Report verifies widespread propagation of the azithromycin resistance gene *mphA* across *Salmonella* populations (13). Given these evolving resistance characteristics, developing novel antibacterial agents capable of penetrating multidrug resistance barriers while preserving precise targeting accuracy represents an urgently required breakthrough strategy.

Phages (>10³¹ particles in nature) represent potent natural antimicrobial agents with significant potential for bacterial biological control (14). Research confirms that phage therapy provides effective control of bacterial contamination in the food chain. Lytic phages offer three key advantages: broad host range, efficient lytic activity, and adaptability to various food matrices. These characteristics are demonstrated through multiple applications, including *Salmonella* control in foods (15–18), combined phage-thermal processing against *Escherichia coli* (19), *Cronobacter sakazakii* management in dairy products (20), and *Aeromonas hydrophila* biofilm removal from produce (21). These findings support the value of phages as practical biocontrol agents for food safety. This modification demonstrates a broad spectrum of phage applications, building a solid field background. Key regulatory breakthroughs include the Food and Drug Administration (FDA) recognition of various lytic phage-based interventions as generally recognized as safe (GRAS), notably exemplified by the GRAS-certified composite phage preparation SalmoFresh (containing six lytic phages) targeting foodborne pathogens—a crucial advancement demonstrating progress toward commercialization (22). Shakeri et al. isolated and characterized phage vB_StyS-LmqsSP1, specific for lysing the *S*. Typhimurium prevalent in poultry intestines and confirmed its efficacy in significantly reducing target *Salmonella* populations on chicken skin (23). Compared to conventional antibiotics, bacteriophages offer targeted lysis against specific pathogens, exhibit an extremely low tendency to induce resistant mutants, have a minimal impact on host gut microecological balance, and thus provide a sustainable solution for combating antibiotic-resistant bacteria (24).

This study aims to isolate bacteriophages from slaughterhouse and farm wastewater, employing the double-layer agar assay to select a phage isolate demonstrating high lytic activity, a broad host range, and stability against multiple common *Salmonella* serotypes. This isolate will subsequently undergo comprehensive genomic annotation and phylogenetic analysis. Furthermore, we will rigorously evaluate its antibacterial efficacy within various food matrices (e.g., dairy, meat, and eggs) and on biofilm-coated surfaces, particularly assessing its biofilm prevention and removal efficacy on the food-contact materials PP and PE. Ultimately, this research will enhance the diversity of the *S*. Typhimurium phage repository and provide a crucial theoretical foundation for the development of phage-based biocontrol strategies. These strategies target controlling *Salmonella* contamination in food and inhibiting its biofilm formation on food-contact surfaces.

## MATERIALS AND METHODS

### Bacterial strain revival and sample collection

The bacterial strains used in this study included *S*. Typhimurium CMCC 50115 obtained from the National Center for Medical Culture Collections, as well as additional strains preserved by Gansu Agricultural University. Strains were recovered from–80°C glycerol stocks, streaked onto xylose lysine deoxycholate (XLD) agar plates, and incubated at 37°C for 12 h. Glossy or fully black colonies were selected to inoculate lysogeny broth (LB) for overnight culture with shaking at 37°C prior to experiments. Concurrently, between March and October 2023, 115 environmental samples (effluent, silage, and manure) were collected from cattle, yak, pig, sheep, and poultry livestock facilities across Gansu Province for phage isolation.

### Isolation, purification, and preparation of phages

*S*. Typhimurium strain CMCC 50115 served as the host bacterium and was cultured to logarithmic growth phase (OD_600_ = 0.6). An enrichment culture for phage amplification was established by mixing the bacterial suspension, filtered sample, and LB medium at a 1:2:4 volumetric ratio, followed by overnight incubation at 37°C with shaking at 200 rpm. After incubation, 5 µL of the enrichment was spotted onto pre-prepared double-layer agar plates consisting of an LB base layer solidified with 1.5% agar and an overlay of LB containing 0.5% agar, inoculated with the host bacterium. These plates were incubated at 37°C for 6–8 h to facilitate plaque development. Distinct plaques exhibiting transparency and well-defined edges were identified for further processing. The enrichment was then subjected to serial dilution, and 100 µL of an appropriately diluted aliquot was gently mixed with an equal volume of host bacterial suspension (OD_600_ = 0.6) and incubated at 37°C for 10 min. Add this mixture to pre-warmed 3 mL upper-layer soft agar (0.5% agar LB) and mix thoroughly. Rapidly pour onto the lower layer plate and spread evenly. Incubate at 37°C overnight. Observe results the following day. Using a Pasteur pipette, aspirate individual clear, translucent plaques with well-defined edges and store them in SM buffer.

### Transmission electron microscopy (TEM)

Phages were pelleted by centrifugation (10,000 rpm, 1 h), resuspended in 0.1 mol/L ammonium acetate, and adsorbed onto TEM copper grids for 5 min. After negative staining with phosphotungstic acid solution for 10 min, the samples were examined using a Hitachi H-7000FA TEM (Tokyo, Japan).

### Host range

We performed a preliminary assessment of the phage host range against a library of 72 *Salmonella enterica* wild-type strains using the spot assay. Purified phages were diluted in SM buffer to a concentration of 10^9^ PFU/mL, and 5 µL aliquots of the lysate were spotted onto top agar plates containing the respective *Salmonella* host bacteria. Lytic activity was semi-quantitatively scored on a 0–4 scale, and the numerical features of the plaques were further analyzed using hierarchical clustering based on Euclidean distance, where 0 represents no lysis and four indicates complete clearing, after incubation at 37°C for 8 h. The resulting raw data were standardized and subsequently visualized as a heatmap to delineate the phage host range profile.

### MOI

To quantify bacteriophage infectivity, multi-step serial 10-fold phage dilutions were co-cultured with host bacteria at MOI ratios ranging from 0.0001 to 100. Following 3 h of co-incubation, the bacterial cultures were centrifuged (10,000 rpm, 30 min, 4°C) to harvest phage-adsorbed cells, with the supernatants carefully aspirated. The supernatants were resuspended in LB broth, and phage titers were quantified using the double-layer agar technique. Each MOI condition underwent triplicate testing, and the average phage efficacy values were utilized to determine the optimal infection multiplicity.

### Biological characterization of phage W5

#### One-step growth curve

The experimental procedure involved mixing 100 μL of bacteriophage suspension (1 × 10^9^ PFU/mL) with 100 μL of CMCC 50115 host bacterial suspension (1 × 10^9^ CFU/mL) to achieve an MOI of 1, followed by static incubation for 20 min to facilitate phage adsorption. After centrifugation at 12,000 rpm for 2 min, the supernatant was discarded, and the bacterial pellet was resuspended in 50 mL of fresh LB medium for incubation at 37°C, with shaking at 200 rpm. During the 120-min infection period, 200 μL samples were collected at 10-min intervals and centrifuged at 12,000 rpm for 30 s to remove cellular debris. The resulting supernatant 100 μL was then mixed with an equal volume of indicator bacteria and subjected to plaque assay using the double-layer agar method to determine phage titer. Burst Size = (Final PFU − Unadsorbed PFU)/(N₀ × Adsorption Efficiency), where Final PFU is the final progeny phage titer (PFU/mL) after the burst and N_0_ is the initial concentration of host cells (CFU/mL) (25).

#### Thermal and pH stability of phage W5

Phage thermal stability was assessed as described previously (16). Samples were incubated at 10°C–80°C in LB medium for 60 min, and then, residual activity versus host bacteria was determined via double-layer agar plaque assay to derive the thermal stability profile. For pH stability, phage W5 was diluted in SM buffer (pH 2, 4, 6, 7, 8, 10, 12, and 13), incubated at 37°C for 1 h, mixed with host bacteria at the optimal MOI, and the resulting phage titer was measured to identify the optimal pH range.

#### Adsorption rate of phage W5

A host bacterial culture in the logarithmic growth phase is mixed with a bacteriophage suspension at an MOI of 1 and incubated statically at room temperature. At 2, 4, 6, 8, 10, 15, and 20 min post-infection, 100 µL aliquots of the mixture are collected and immediately centrifuged at 12,000 rpm for 1 min to pellet bacteriophage-bacterial complexes. The supernatant is recovered, and the residual titer of unadsorbed bacteriophages is quantified via the double-layer agar plaque assay. The number of adsorbed bacteriophages at each time point is calculated using the formula: adsorbed phages = initial total phage count − residual phage titer in supernatant. This value is then converted to adsorption percentage: (Adsorbed Count/Initial Count) × 100%.

#### Effect of organic solvents on phage activity

To evaluate the effects of various organic solvents on phage activity, a phage solution with an initial titer of 1 × 10^9^ PFU/mL was mixed separately with equal volumes of isoamyl alcohol, glycerol (propane-1,2,3-triol), methanol, ethanol, chloroform, and SM buffer. Each mixture was incubated at 37°C for 15 min. After incubation, the titer of the phage suspension in each treatment group was measured.

### Characterization of the phage W5 genome

A 100 µL aliquot of bacteriophage suspension was mixed with an equal volume of host bacterial culture and incubated at 37°C for 15 min to allow adsorption, after which the mixture was transferred into 100 mL of LB medium for shake-cultivation. Post-culture supernatant was collected and centrifuged (4°C, 8,000 rpm, 15 min), followed by filtration through a 0.22 µm membrane; DNase I and RNase I were added to the filtrate at a final concentration of 1 µg/mL each for incubation at 37°C for 50 min to degrade free nucleic acids. Subsequently, NaCl was added to a final concentration of 1 mol/L, and the solution was incubated on ice for 1 h before precipitated impurities were removed by repeated centrifugation (4°C, 8000 rpm, 10 min). Phages were precipitated from the supernatant by adding 10% (wt/vol) PEG 8000, collected by centrifugation (4°C, 10,000 rpm, 15 min), and the resultant pellet resuspended in 1 mL SM buffer with incubation at room temperature for 12–14 h to facilitate phage particle dissolution. Following the addition of 1 mL chloroform for liquid-liquid extraction and centrifugation (4°C, 5000 rpm, 15 min), the upper aqueous phase was collected; chloroform extraction was repeated 1–2 times as required for purity, and the final aqueous phase was filter-sterilized through a 0.22 µm membrane to obtain concentrated phage lysate. Genomic DNA was extracted from this concentrate and submitted to FSGene Sciences & Technology (Wuhan, China) for whole-genome sequencing on the PacBio Sequel II platform. Bioinformatic analysis included mapping raw sequencing reads against a reference genome using BWA (v0.7.12), extracting mapped sequences for genome assembly and coding sequence (CDS) prediction via Prodigal, and performing BLAST (v2.2.31+) alignment of predicted protein sequences against databases for gene annotation based on optimal E-values and sequence similarity thresholds. Subsequent analyses entailed phylogeny reconstruction of the phage terminase large subunit using MEGA 11.0, genomic synteny assessment with Easyfig, and whole-genome average nucleotide identity (ANI) calculation employing Pyani software.

### Biological control of *S*. Typhimurium in foods using phage W5

Fresh milk samples were aseptically processed and inoculated with CMCC 50115 to achieve a final concentration of 10^3^–10^4^ CFU/mL in 5 mL systems. Market-sourced swine carcass meat was cut into cylindrical sections, 1 cm in diameter and 0.5 cm thick, sterilized via dual-side UV irradiation for 30 min per side, inoculated with an equivalent bacterial concentration, and air-dried for 15–20 minutes within a biosafety cabinet (17). Commercially purchased shell eggs underwent surface decontamination; they were sequentially disinfected with 75% ethanol, rinsed three times with sterile water, dried with sterile filter paper, and irradiated under UV light for 30 min (26). Whole egg liquid was aseptically separated and homogenized, while the inner shell surface was scraped, both then being inoculated with an equivalent *S*. Typhimurium load. All experimental groups received bacteriophage suspensions with multiplicity of infection values of either 1 or 100, whereas control groups received equivalent volumes of phosphate-buffered saline (PBS) buffer. All samples were subsequently incubated under both 4°C and 30°C conditions. Sampling occurred at 0, 4, 8, 12, 24, and 48 h time points for analysis. Milk samples underwent direct serial dilution and spread plate counting. Pork samples were mixed with 5 mL PBS buffer, processed initially using a high-speed homogenizer (10,000 rpm for 2 min), followed by ultrasonic treatment (99 W power at 40 kHz frequency for 5 min). Liquid eggs and eggshell samples were processed separately using oscillation elution methods. Pathogen load in all samples was quantified via plate counting on *Salmonella*-specific chromogenic medium incubated at 37°C for 24 h.

### Evaluation of the inhibitory effect and removal of *S*. Typhimurium biofilm by phage W5

This study systematically evaluated the anti-biofilm efficacy of bacteriophage W5 utilizing a dual strategy combining biofilm formation inhibition and mature biofilm eradication. For the biofilm formation inhibition assay, employing a modified method derived from Cao et al. (27), 100 µL of CMCC 50115 suspension (OD_600_ = 0.2) was added to 96-well plates. Control groups were supplemented with 200 µL of LB liquid medium, while experimental groups had 100 µL of W5 phage suspension at MOI = 1 or MOI = 100 mixed in. Plates were incubated at 37°C for 24, 48, and 72 h with measurements taken at designated time intervals. Following incubation, the culture medium was removed, and adherent cells underwent three to five washes with 200 µL of sterile PBS. Plates were then dried at room temperature, and biofilms were fixed using 95% methanol for 15 min. After three PBS washes and air-drying, 250 µL of 1% crystal violet staining solution was added to perform 30 min of staining at room temperature. Unbound dye was removed by rinsing under running water, and the plates were dried. Finally, bound dye was released through 15 min of agitation with 250 µL of 95% anhydrous ethanol. Absorbance at OD_595_ was quantified using a microplate reader. Regarding mature biofilm eradication, based on an optimized protocol from Webber et al., 100 µL of *Salmonella* suspension (OD_600_ = 0.2) was first pre-cultured at 37°C for 72 h to establish biofilms (28). Control groups received 200 µL of LB medium, whereas experimental groups were treated with 100 µL of bacteriophage suspension at MOI = 1 for an additional 24–72 h. Crystal violet staining and OD_595_ absorbance measurement were subsequently performed following the identical protocol described above.

### CLSM

To evaluate the inhibitory effect of phage W5 on *S*. Typhimurium biofilms at different developmental stages under 4°C and 30°C, we employed CLSM coupled with live/dead cell dual staining. Specifically, exponential-phase bacterial cultures were inoculated into 20-mm confocal dishes and incubated at the target temperatures for 24 h to form young biofilms and for 72 h to establish mature biofilms. The biofilm samples were subsequently treated with phage W5 at their respective temperatures for 24 h. Post-treatment, planktonic cells were removed by PBS rinsing, and the retained biofilms were stained in the dark with 10 µM SYTO9 and 30 ABCD-2M propidium iodide (PI) at their original culture temperatures for 25 min. After a secondary PBS wash, fluorescence images were acquired using CLSM, followed by analysis.

### FESEM

The effect of phage W5 on *S*. Typhimurium biofilms on food contact material surfaces was evaluated using FESEM. PE and PP slides (2 × 2 × 0.01 cm) were pretreated with 70% ethanol, sterilized, and dried. Log-phase bacterial cultures were inoculated onto the substrate surfaces. Young biofilms were formed by incubating the substrates at 4°C and 30°C in 12-well plates for 24 h, or mature biofilms were formed by incubating the substrates at 30°C for 72 h (with LB medium replaced every 24 h). After rinsing with PBS to remove free-floating bacteria, the plates were treated with phage W5 at an infection multiple (MOI) of 100 for 8 h at the same temperature. Transfer the slides to tubes containing 10 mL of PBS and 20 glass beads, vortex to dissociate the biofilm, dilute the suspension with PBS, spread on LB agar, and incubate at 30°C for 24 h for counting. FESEM samples were sequentially fixed at room temperature with 2.5% glutaraldehyde (Sigma-Aldrich) for 2 h, washed three times with PBS, fixed in 1% osmium tetroxide in the dark for 2 h, dehydrated with a gradient of ethanol (30%, 50%, 70%, 90%, and 100%), air-dried for 3 h, and gold-coated. The samples were observed using a field emission scanning electron microscope (Carl Zeiss, Germany) at an acceleration voltage of 5 kV and a working distance of 15 mm.

### Statistical analysis

All experiments were independently conducted with three repetitions. Data were statistically analyzed using GraphPad Prism 8.2.1 software (GraphPad Software, La Jolla, CA, USA). The results are expressed as means, with error bars in the figures representing the Standard Error of the Mean (SEM). Two-way analysis of variance (ANOVA) was performed on the obtained data with a 95% CI, while within-group variation analysis employed one-way ANOVA. Statistical significance between treatment groups was set at *P* < 0.05, specifically denoted as follows: *P* < 0.05; *P* < 0.01; *P* < 0.001; non-significant (ns): *P* > 0.05.

## RESULTS

### Isolation, screening, and host range determination of phages

**Fig 1:**
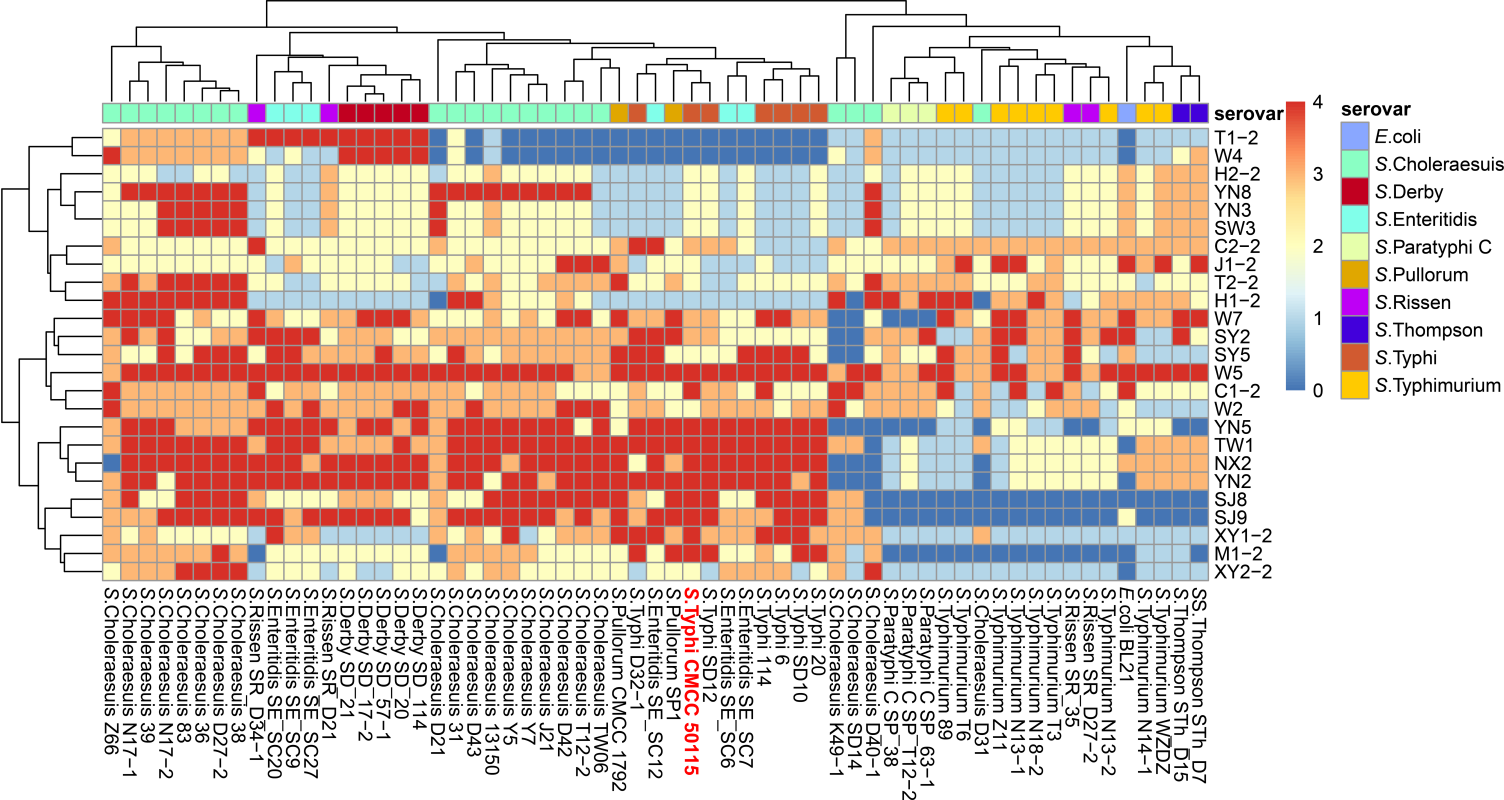
Host range cluster analysis with lytic activity profiles of isolated phages. Lytic activity was semi-quantitatively scored: 0 (dark blue, no lysis), no plaque formation; 1 (light blue, weak), minimal plaque formation with turbid zones; 2 (white, partial), distinct but turbid halos; 3 (light red, significant), near-clear zones with residual turbidity; 4 (dark red, complete), well-defined transparent plaques.

**Fig 2:**
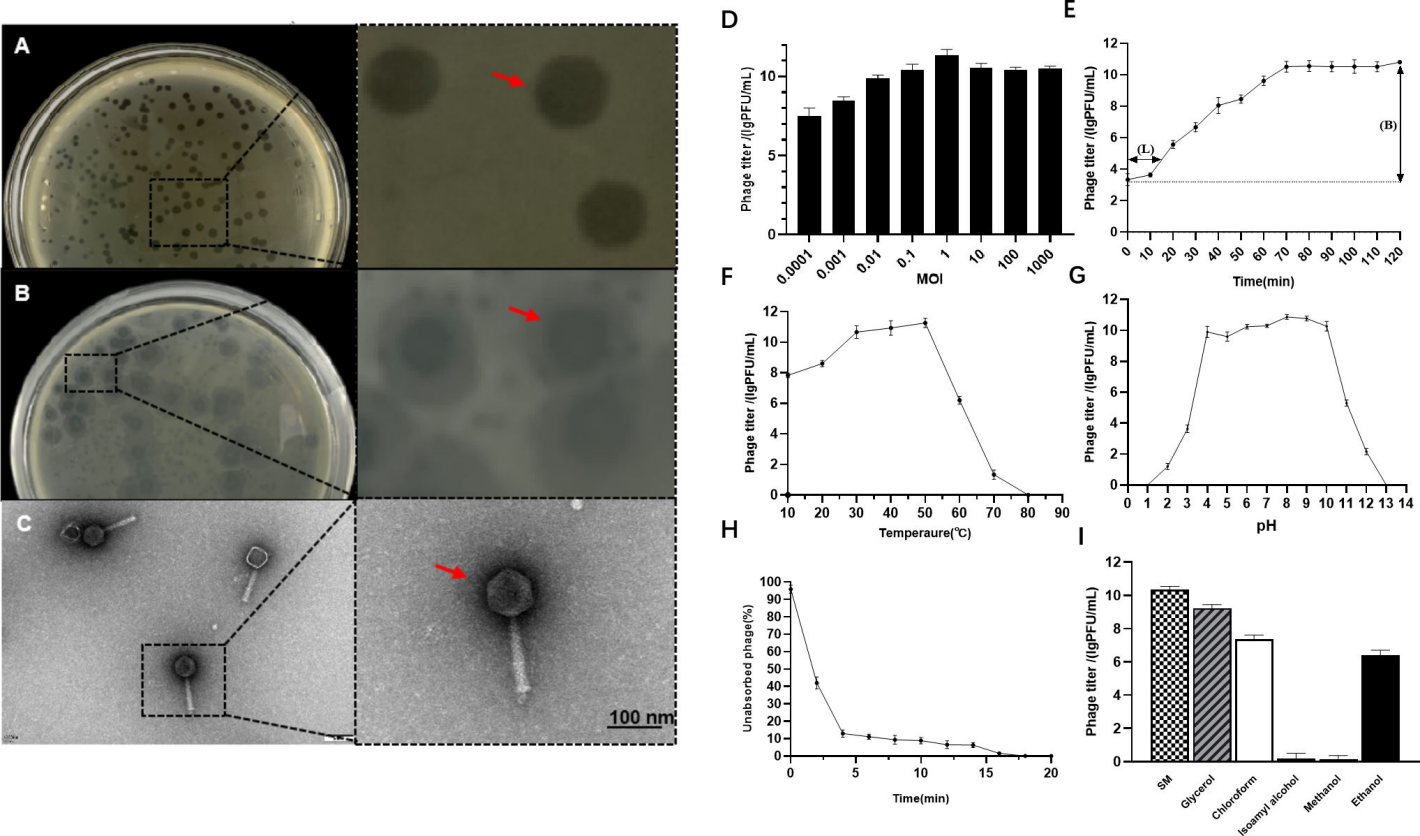
Morphological characterization and biological properties of phage W5. (**A**) Isolation and purification plate (24 h incubation). (**B**) Halo zone formation (72 h incubation). (**C**) Representative TEM image. (**D**) Multiplicity of infection (MOI). (**E**) One-step growth curve. (**F**) Thermal stability. (**G**) pH stability. (**H**) Adsorption kinetics. (**I**) Stability in organic solvents.

Twenty-five *Salmonella*-specific phages were isolated from slaughterhouse/livestock farm wastewater, with phage W5 demonstrating the broadest host range. This phage efficiently lysed—including *S*. Pullorum, *S*. Typhimurium, and *S*. Enteritidis—and maintained high lytic activity against the MDR *Salmonella* strains CMCC 50115 (Table S1). Given its potent elimination capacity against MDR *Salmonella*, W5 was selected as a candidate for development into a novel bacteriophage-based biocontrol strategy targeting *Salmonell*a contamination in food processing environments (Fig. 1). TEM observation revealed that bacteriophage W5 exhibits typical *Myoviridae* morphological characteristics: a polyhedral head structure (diameter 115 ± 2 nm) and a contractile syringe-like tail structure (length 200 ± 2 nm) (Fig. 2A through C).

### Biological properties of phage W5

A study on the biological characteristics of phage W5 demonstrated that its infection efficiency correlates with multiple environmental factors. Host bacteria infected under different MOI conditions showed that MOI = 1 was the optimal multiplicity of infection, yielding the highest titer of 1.69 × 10^11^ PFU/mL (Fig. 2D). Growth curve analysis (MOI = 1) revealed that the phage titer remained relatively stable during the first 20 min, then entered an exponential growth phase (20–70 min), peaking at 3.8 × 10^11^ PFU/mL at 70 min (Fig. 2E). Phage W5 maintained high titer levels within the temperature range of 10°C–50°C, with infectious activity ranging from 5.0 × 10^8^ to 1.4 × 10^11^ PFU/mL. At 80°C, the phage completely lost infectivity, confirming its significant temperature-dependent biological activity as stable under moderate-to-low temperatures but extremely sensitive to high temperatures, which provides critical guidance for transportation, storage, and inactivation in practical applications (Fig. 2F). pH-tolerance experiments demonstrated that W5 remained stable within pH 4–11 (Fig. 2G), with activity dropping sharply beyond this range. Phage W5 exhibited rapid and efficient adsorption, reaching 89% binding efficiency within 4 min post-infection, exceeding 98% by 15 min, and achieving 98% adsorption within 13 min (Fig. 2H). W5 showed differential sensitivity to organic solvents: isoamyl alcohol and methanol caused complete inactivation, while SM buffer and glycerol maintained relatively high titers (1.4 × 10^8^–5.7 × 10^10^ PFU/mL). Chloroform and ethanol led to partial inactivation (Fig. 2I).

### Genomic characterization of phage W5

**Fig 3:**
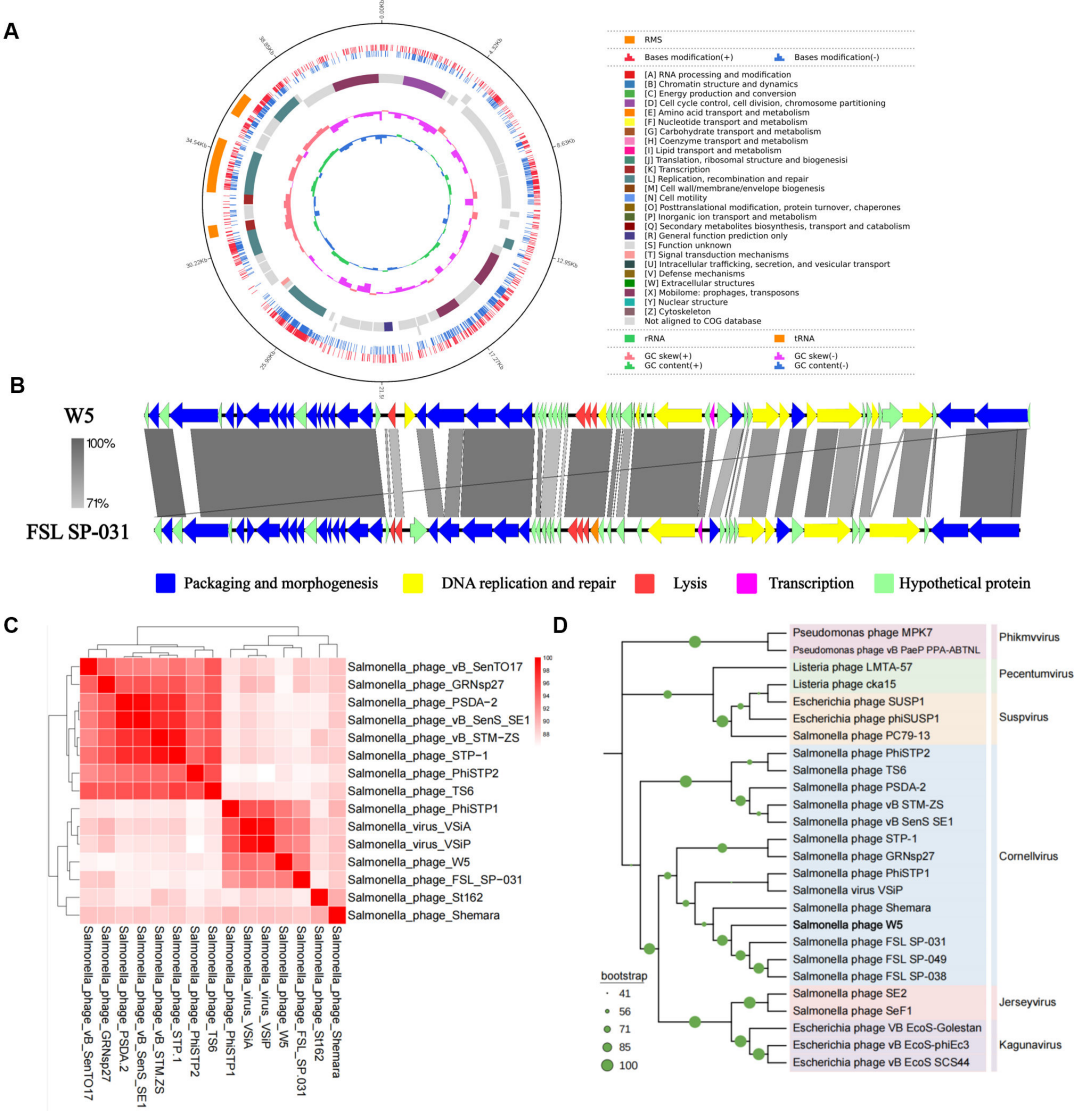
Genomic characterization of phage W5. (**A**) Circular genome map of W5. (**B**) Synteny alignment showing homology with phage FSL SP-031 (arrows indicate predicted CDSs). (**C**) ANI heatmap comparing 15 *Guernseyvirinae* phages. The color gradient represents whole-genome similarity (88%–100%), with the dashed line indicating the 95% threshold commonly used for species demarcation. (**D**) Phylogenetic tree based on the large terminase subunit (total branch length = 0.31527374, bootstrap support >90%).

Genomic analysis of *S*. Typhimurium phage W5 reveals typical characteristics of the order *Caudovirales*, with a genome length of 43,714 bp and a GC content of 51.41% (Fig. 3A). Functional annotation identified CDSs accounting for 52% of the genome, including key functional genes encoding structural proteins, DNA replication-related enzymes, and lytic enzymes. Synteny analysis generated using Easyfig v.2.0 (Fig. 3B) demonstrated highly conserved gene organization and functional modules between W5 and the closely related phage FSL SP-031 (NC_021775.1), with particularly significant BLAST homology observed in DNA replication and structural protein regions. ANI heatmap analysis (Fig. 3C) further revealed ANI values ranging from >88% between W5 and 15 members of the *Guernseyviridae* family. Phylogenetic analysis based on the large terminase subunit gene (Fig. 3D) definitively classified W5 as a member of the *Cornellvirus* genus, forming a well-supported clade (bootstrap value >90%) with FSL SP-031. The maximum composite likelihood analysis yielded a total branch length sum of 0.31527374 and a substitution rate of 0.315 substitutions per site. Collectively, these findings demonstrate that phage W5 represents a novel member of the *Cornellvirus* genus, and its genomic architecture and phylogenetic characteristics provide crucial insights for future investigations into host adaptation mechanisms and evolutionary dynamics within this taxonomic group.

### Application of phage W5 in controlling *S*. Typhimurium in foods and biofilms

To assess the phage-mediated biocontrol of S. Typhimurium in food matrices, pasteurized milk, raw pork, liquid whole egg, and eggshell surfaces were artificially inoculated. As shown in Table 1 (4°C) and Table 2 (30°C), significant bactericidal effects (*P* < 0.01) were observed across all phage-treated groups (MOI = 1 and 100) in all tested foods. Overall, the treatment with MOI = 100 demonstrated stronger antibacterial efficacy than MOI = 1, and the potency varied among different food matrices. For instance, the highest bacterial reductions (approximately 3.5–3.9 log) were observed in liquid whole egg and on eggshell surfaces, while a relatively weaker effect was seen in raw pork. These results indicate that phage W5 can significantly suppress antimicrobial-resistant *Salmonella* contamination in multiple food matrices, demonstrating practical biocontrol potential.

**TABLE 1:** Biocontrol efficacy of phage W5 against *S*. Typhimurium in different food matrices at 4°C

Food matrix	Reduction at MOI = 1 (log)	Reduction at MOI = 100 (log)	Time to significant effect (h)
Pasteurized milk	2.093	3.897	4
Raw pork	1.687	2.687	8
Liquid whole egg	3.473	3.607	8
Eggshell surface	3.137	3.697	8

**TABLE 2:** Biocontrol efficacy of phage W5 against *S*. Typhimurium in different food matrices at 30°C

Food matrix	Reduction at MOI = 1 (log)	Reduction at MOI = 100 (log)	Time to significant effect (h)
Pasteurized milk	2.330	3.917	4
Raw pork	2.690	2.773	8
Liquid whole egg	3.677	3.690	8
Eggshell surface	3.327	3.717	8

**Fig 4:**
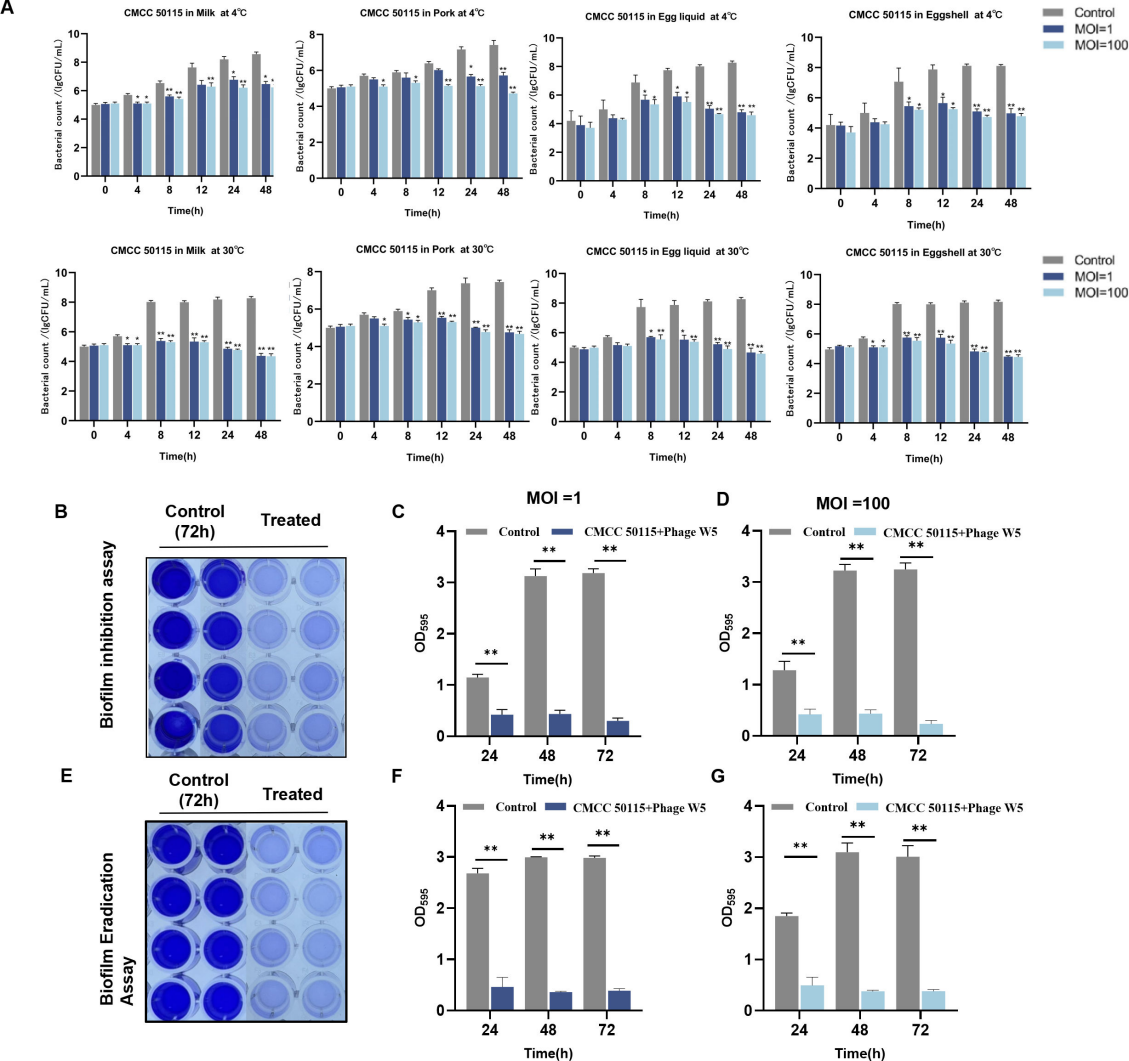
Biocontrol effect of phage W5 on CMCC 50115 in different food matrices. (**A**) Biocontrol efficacy of phage W5 against CMCC 50115 in milk, raw pork, and eggs (shell and liquid) at 4°C and 30°C. (**B**) Inhibition of biofilm formation by phage W5. A 100 µL suspension of CMCC 50115 was added to 96-well plates. The control group was supplemented with 200 µL of LB liquid medium, while the experimental groups received 100 µL of phage W5 suspension at MOI = 1 or MOI = 100. After incubation at 37°C for 72 h, residual biofilm biomass was quantified by crystal violet staining, and absorbance was measured at 595 nm using a microplate reader to evaluate the inhibitory effect. (**C and D**) Inhibitory effect of phage W5 treatment (MOI = 1 and 100) on biofilm formation over 72 h. (**E**) Eradication of preformed biofilms by phage W5. Mature biofilms of CMCC 50115, developed over a 72-h incubation period, were treated in 96-well plates with phage W5 suspensions at MOI = 1 and 100 at 37°C for 72 h. Post-treatment, residual biofilm biomass was quantified by crystal violet staining, and absorbance was measured at 595 nm using a microplate reader to assess eradication efficacy. (**F and G**) Effect of phage W5 treatment (MOI = 1 and 100) in removing mature biofilms over 72 h. Data were analyzed by one-way ANOVA followed by Tukey’s post hoc test (ns, not significant; *P < 0.05; **P < 0.01; *P < 0.001).

Using a 96-well plate crystal violet assay, we assessed the *in vitro* biofilm formation capacity of *S*. Typhimurium CMCC 50115 (Fig. 4B). The optimal biofilm formation occurred at 72 h of incubation; therefore, this time point was selected to evaluate phage W5’s effects on biofilm formation and eradication (Fig. 4E). When challenged with phage W5 (10^8^ PFU/mL), antimicrobial-resistant *Salmonella* exhibited significantly suppressed biofilm formation at both 48 h and 72 h at OD_595_ nm (Fig. 4C and D). Similarly, mature biofilms at 72 h post-incubation showed significant removal upon phage treatment at OD_595_ nm (Fig. 4F and G). These results demonstrate phage W5’s dual inhibitory function in preventing nascent biofilm development and disrupting established biofilms.

### CLSM assessment of anti-biofilm activity by phage W5

**Fig 5:**
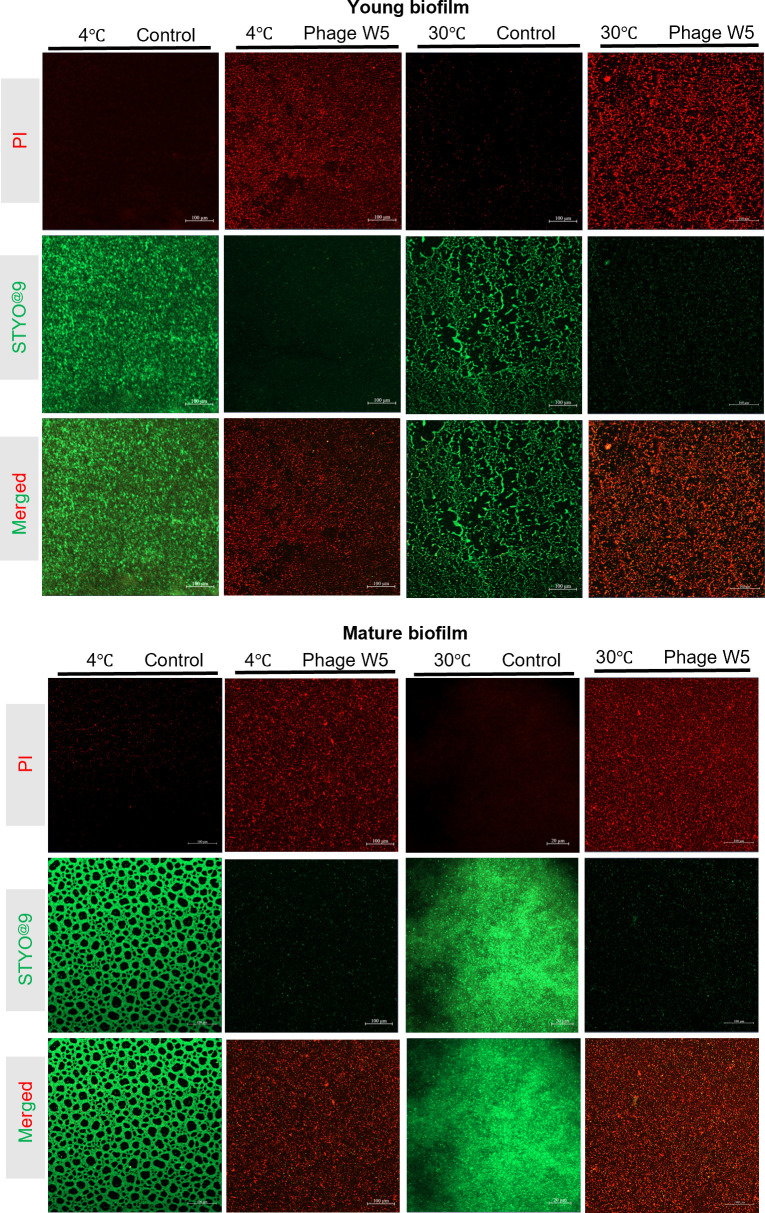
Bacteriophage-mediated eradication of biofilms. CLSM analysis was employed to quantify the clearance efficacy of phages against *S*. Typhimurium CMCC 50115 biofilms under dual-variable conditions: cultivation temperature (4°C/30°C) and biofilm maturity stages (young biofilm: 24 h; mature biofilm: 72 h). Following dual-staining with SYTO 9 (green fluorescence labeling viable bacteria) and PI (red fluorescence labeling dead bacteria), microbial elimination efficiency was imaged and quantified based on live/dead bacterial fluorescence signals.

CLSM analysis demonstrated that phage W5 effectively eliminated both early-stage and mature biofilms of *S*. Typhimurium CMCC 50115 at different cultivation temperatures. SYTO 9-generated green fluorescence identified viable bacteria, while PI red fluorescence and yellow signals formed by overlapping red/green emissions indicated membrane-damaged or dead cells. Control biofilms at 4°C and 30°C exhibited persistently dense green fluorescence, confirming physiologically intact bacterial states (Fig. 5); in contrast, phage W5 treatment caused complete biofilm conversion to a dominantly red fluorescence pattern across all regions, with the most pronounced effect observed at 30°C. This eradication efficacy originated from synergistic interaction between the phage’s superior matrix penetration capacity and temperature-dependent lytic activity, significantly enhancing bacterial clearance efficiency within biofilms.

### FESEM evaluation of phage W5 inhibitory effects on PE/PP biofilms

**Fig 6:**
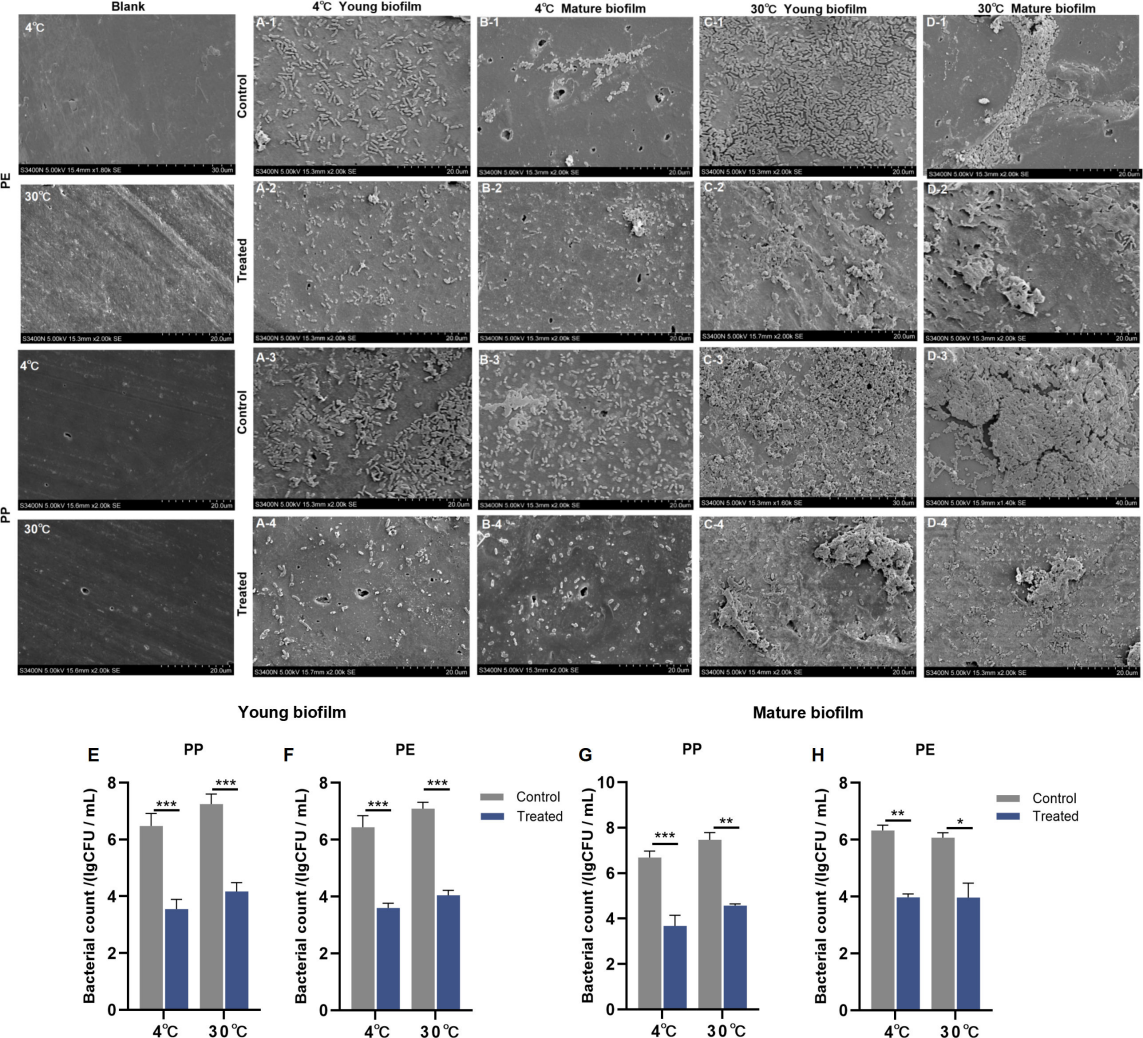
The efficacy of phage W5 (MOI 100) in removing S. Typhimurium CMCC 50115 biofilms on food-contact material surfaces. (**A–D**) FESEM images of biofilms on material surfaces (scale bar: 20 µm, ×1,000): (A1–2) Young biofilms formed on PE at 4°C after phage treatment. (A3–4) Young biofilms formed on PP at 4°C after phage treatment. (B1–2) Mature biofilms formed on PE at 4°C after phage treatment. (B3–4) Young biofilms formed on PP at 4°C after phage treatment. (C1–2) Young biofilms formed on PE at 30°C after phage treatment. (C3–4) Young biofilms formed on PP at 30°C after phage treatment. (D1–2) Mature biofilms formed on PE at 30°C after phage treatment. (D3–4) Mature biofilms formed on PP at 30°C after phage treatment. (**E–H**) Quantitative analysis of phage removal efficiency on young (**E, F**) and mature (**G, H**) biofilms on PP and PE. **Data were analyzed by one-way ANOVA followed by Tukey’s post hoc test (ns, not significant; *P < 0.05; **P < 0.01; *P < 0.001; ***P < 0.001).

FESEM images clearly demonstrate morphological differences in *S*. Typhimurium biofilms (young/mature) on PE and PP surfaces before and after phage W5 treatment, as shown in Fig. 6. Blank control surfaces (without biofilm) showed scarce cracks and significantly higher smoothness (Fig. 6A and B). In control groups, numerous *S*. Typhimurium CMCC 501115 cells tightly adhered to PE and PP surfaces, forming highly cohesive dense structures. Notably, biofilm formation displayed significant temperature dependence: only loose, sparsely distributed biofilms formed at 4°C (Fig. 6A–1, 3, B-1 and 3), while dense aggregates with high bacterial density developed at 30°C (Fig. 6C–1, 3, D–1 and 3). After phage W5 treatment, biofilms on both PE and PP surfaces underwent significant disintegration, with only a few planktonic cells remaining; originally intact compact biofilms exhibited large-area damage, and substantial bacterial cells showed characteristic perforations and membrane structural damage (Fig. 6ABCD-2 and ABCD-4). Consistent with biofilm morphological disruption, quantitative results of phage W5 (MOI 100) inhibitory effects on *S*. Typhimurium biofilms formed on PP and PE at 4°C and 30°C demonstrated that upon phage treatment, young biofilms and mature biofilms on PP decreased by 2.933 log CFU/mL and 3.080 log CFU/mL at 4°C, and by 3.010 log CFU/mL and 2.907 log CFU/mL at 30°C, respectively (Fig. 6E and G). Similarly, young biofilms and mature biofilms on PE decreased by 2.840 log CFU/mL and 3.050 log CFU/mL at 4°C, and by 2.350 log CFU/mL and 2.107 log CFU/mL at 30°C, respectively (Fig. 6F and H). The eradication efficacy of W5 varied depending on biofilm maturity, material surface, and temperature conditions. Notably, the highest reduction in young biofilms was observed on PE at 30°C, while the most effective clearance of mature biofilms occurred on PP at 4°C.

## DISCUSSION

In recent years, bacteriophages have garnered significant attention in food safety due to their unique antibacterial mechanisms. The bacteriophage W5 isolated in this study demonstrates remarkable host specificity, efficiently lysing nine major pathogenic *Salmonella* serotypes, including *S*. Typhimurium, *S*. Typhi, *S*. Pullorum, and *S*. Choleraesuis. Compared with the previously reported *Salmonella* phage LPST153, W5 maintains sustained suppression of host growth for up to 12 h even at a low MOI of 0.1 while retaining high lytic activity (29). Phage W5 exhibits an exceptionally short latent period of 15 min and a high burst size exceeding 100 PFU/cell. This rapid proliferation characteristic makes it particularly effective in food processing environments, as studies have confirmed that phages with shorter latent periods can significantly reduce viable bacterial concentrations on food contact surfaces and processing equipment, as demonstrated (30). Thermostability and pH tolerance are crucial phenotypic traits influencing the efficacy of phage-based biocontrol applications. Phage W5 maintains high activity across a broad pH range of 3–13 and remains stable for at least 120 minutes at 55°C. Compared with other reported *Salmonella* phages as documented by Hosny et al. (31, 32), W5 exhibits superior tolerance to extreme environmental conditions, providing distinct advantages for its application in complex settings such as food processing or medical disinfection. Organic solvent screening is critical not only for preserving phage structural integrity, including capsid protein conformational stability, but also for optimizing the loading efficiency of delivery systems, as emphasized previously (27). This study found that W5 retains over 90% infectious titer in both SM buffer and propylene glycol, showcasing dual-phase solvent compatibility, which confers unique advantages for its incorporation into food matrices. Propylene glycol, as a GRAS-grade solvent, ensures food safety, while the SM buffer serves as a stable medium for industrial freeze-drying processes. Collectively, the broad-spectrum antibacterial activity and robust stability of W5 underscore its potential for commercialization as a biopreservative, addressing key technical challenges in controlling pathogenic bacterial contamination across the food supply chain while ensuring high bactericidal efficiency alongside compatibility with existing processing technologies and regulatory safety standards.

The genomic evolutionary trajectory of *Salmonella* phage W5 operates in deep synergy with its endolysin mechanism, collectively constituting a dual safeguard for highly efficient pathogen control. Collinearity analysis further reveals that W5 forms a highly conserved cluster of gene module clusters with other *Salmonella* phages such as FSL SP-031 and VSiP (Fig. 3B). The genetic fixation of their core functional domains (e.g., tail fiber protein and DNA packaging enzymes) likely stems from long-term host-phage co-evolution. Notably, the W5 endolysin is consistently localized within homologous genomic regions across related phages. Its conservation spanning multiple serotypes can be attributed to adaptive optimization targeting the fundamental structure of the host cell wall throughout evolution. Critically, its receptor-binding protein (RBP) achieves broad-spectrum coverage against diverse serotypes by recognizing varied host surface receptors (e.g., outer membrane proteins/lipopolysaccharides). This represents the core mechanism enabling W5 to overcome serotype limitations (33–35). Furthermore, the W5 endolysin exhibits unique advantages for food safety applications. First, its targeting of invariant peptidoglycan backbone structures mitigates the risk of efficacy loss due to host mutations. Second, its enzymatic hydrolysis process specifically recognizes β-1,4-glycosidic bonds within the cell wall, selectively degrading bacterial structures while preserving food protein and lipid components. This effectively eliminates the indiscriminate residual contamination commonly associated with chemical disinfectants (36). Most significantly, its enzymatic degradation products are natural oligosaccharide fragments, conforming to the GRAS substance standard for food safety (37). This enables the concurrent achievement of dual objectives, highly efficient disinfection, and safe zero-residue outcomes in scenarios like meat processing. In the future, phage lysins are anticipated to emerge as a novel avenue for the research and development of antibacterial drugs. Certain lysins have been proven to exhibit high bactericidal activity against gram-negative pathogens, including *Pseudomonas aeruginosa* and *Klebsiella pneumoniae* (38). Significantly, with the advent of DeepLysin, the first artificial intelligence (AI) platform designed to mine efficient lytic enzymes from the vast "dark matter" of uncharacterized phage genomes, the lysozyme candidate LLysSA9 has been successfully identified, demonstrating optimal activity within its category and showing remarkable efficacy in animal infection models. These findings suggest that the highly active antimicrobial protein candidates mined by DeepLysin hold substantial potential in the prevention and control of clinically epidemic pathogens, particularly ESKAPE pathogens (39, 40). Collectively, W5, leveraging its highly conserved RBP recognition system and precise lytic mechanism, emerges as an ideal biocontrol tool to address the challenge of multi-serotype *Salmonella* contamination. It thereby provides a novel antibacterial strategy to ensure the biosafety of diverse categories of fresh products.

*S*. Typhimurium, a major pathogen of foodborne illnesses, is commonly found in contaminated food products such as dairy, meat, fresh produce, and seafood (41). The formation of biofilms on food-contact surfaces can lead to persistent contamination, posing severe risks to public health (42). In this study, pasteurized milk, fresh pork, liquid whole egg, and eggshell surfaces were selected as representative matrices. Experimental conditions simulated real-world food processing and storage environments: 30°C represented room-temperature exposure (e.g., slaughtering/cutting or egg mixing), while 4°C mimicked cold-chain storage (e.g., refrigeration of meat or egg products during transport and warehousing) (25). The results demonstrated that phage W5 consistently eradicated drug-resistant strains across all matrices. Its temperature adaptability suggested potential mechanisms such as cold-adaptive protein encoding. Notably, W5 exhibited dual “prevention-treatment” anti-biofilm capabilities—synergistically combining extracellular polymeric substance hydrolase activity with quorum-sensing interference to both inhibit nascent biofilm formation and dismantle mature biofilms. Matrix properties significantly influenced efficacy: performance was optimal in protein-rich liquid whole egg, whereas fresh pork required 8 h for observable effects due to myofiber barriers, aligning with reports of phage penetration challenges in raw meat products (43). These findings highlight the need for future development of microencapsulation delivery systems to enhance efficacy in solid foods. A groundbreaking investigation conducted by Yang et al. presented an innovative oral hydrogel microsphere delivery system. This system can augment the viability of phages in the gastrointestinal tract by more than 100-fold. It effectively surmounts the barriers posed by gastric acid and attains remarkable efficacy in bacterial colitis models, thus resolving the crucial challenge associated with oral phage delivery (44). Collectively, this work not only provides an innovative solution for enhancing the safety of cold-chain foods (e.g., poultry/egg products) but also delivers theoretical and technical support for combating drug-resistant *Salmonella* and biofilm contamination in industrial practice.

Material surface properties decisively influence bacterial adhesion and biofilm formation, with pathogens such as *Salmonella* capable of establishing biofilms on diverse food-contact substrates, including stainless steel, PE, wood, glass, PP, and rubber (10). In this study, CLSM imaging verified that bacteriophage W5 achieves complete eradication of both early-stage and mature biofilms of *S*. Typhimurium CMCC 50115 across temperatures from 4°C (representative refrigeration temperature) to 30°C (optimal growth temperature), mediated through the disruption of bacterial membrane integrity (evidenced by universal replacement of SYTO 9 green fluorescence with PI red emission) (Fig. 4A). Peak clearance efficacy occurred at 30°C, attributed to synergistic enhancement of phage lytic activity and biofilm penetration capability under elevated temperatures. FESEM further revealed extensive collapse of *Salmonella* biofilms on PE/PP surfaces following phage treatment (Fig. 6A–3, 4/B–3 and 4), with residual planktonic cells exhibiting characteristic perforated cytomembrane morphology. Quantitative analysis confirmed significant biofilm biomass reduction (2.107–3.080 log CFU/mL). Juvenile biofilm clearance consistently exceeded that of mature biofilms, particularly demonstrating maximal disparity on PE surfaces at 30°C (2.350 vs. 2.107 log CFU/mL reduction). Destructive efficacy was co-modulated by temperature and material surface properties, with complete disintegration of dense biofilm architectures being favored at 30°C, illuminating a collaborative regulatory mechanism between temperature-mediated thermodynamic lysis processes and material interface adsorption effects. These findings align with Byun et al.’s report on the substantial influence of biofilm maturity on phage clearance efficacy (45). However, the super-hydrophobicity of PE facilitates excessive extracellular polymeric substances secretion, forming a physical barrier (mean pore size <90 nm), thereby compromising phage penetration efficiency (46); this likely underpins the relatively lower clearance rates observed for mature biofilms on PE surfaces. However, the depolymerases encoded by phages, which degrade essential biofilm barriers to overcome penetration limitations, offer a promising approach for enhancing the efficacy of biofilm removal. This strategy exhibits broad application prospects in areas such as food (processing/safety), medical (devices and infection control), and industrial sterilization.

### Conclusion

This study isolated a novel lytic bacteriophage designated W5 from slaughterhouse wastewater, demonstrating significant antibacterial performance and biofilm clearance capabilities. The phage efficiently lyses ten major prevalent *Salmonella* serotypes. Its short latent period and high burst size markedly enhance bacterial suppression efficiency. W5 exhibits groundbreaking extreme environmental stability, maintaining potent lytic activity across ultra-broad pH conditions ranging from pH 3–13, under high-temperature environments at 55°C, and within food matrices including milk, pork, and eggs. It concurrently eliminates both early-stage and mature *S*. Typhimurium biofilms, effectively reducing biofilm biomass on PP and PE material surfaces. Whole-genome analysis confirmed the absence of virulence, antibiotic resistance, and lysogenic genes, meeting safety standards for food-grade biological agents. Integrating its advantages in extreme environment tolerance, efficient biofilm removal, and food safety assurance, W5 provides a technological solution with exceptionally high application potential for *Salmonella* control in the food industry and cross-field biofilm management.

## Data Availability

The phage whole genome data have been uploaded to the NCBI database (PP996006). The corresponding data can be obtained from the first author or corresponding author upon request.

## SUPPLEMENTAL MATERIAL

Table S1 aem.01878-25-s0001.docx Antibiotic resistance profiles of *S*. Typhimurium CMCC 50115.

ASM does not own the copyrights to Supplemental Material that may be linked to, or accessed through, an article. The authors have granted ASM a non-exclusive, world-wide license to publish the Supplemental Material files. Please contact the corresponding author directly for reuse.

## References

[1] Ferrari RG, Rosario DKA, Cunha-Neto A, Mano SB, Figueiredo EES, Conte-Junior CA. 2019. Worldwide epidemiology of Salmonella serovars in animal-based foods: a meta-analysis. Appl Environ Microbiol 85:e00591-19. doi:10.1128/AEM.00591-19 31053586 PMC6606869

[2] Zhang H, Pan S, Zhang K, Michiels J, Zeng Q, Ding X, Wang J, Peng H, Bai J, Xuan Y, Su Z, Bai S. 2020. Impact of dietary manganese on intestinal barrier and inflammatory response in broilers challenged with Salmonella Typhimurium. Microorganisms 8:757. doi:10.3390/microorganisms8050757 32443502 PMC7285304

[3] Ngoi ST, Teh CSJ, Chai LC, Thong KL. 2015. Overview of molecular typing tools for the characterization of Salmonella enterica in Malaysia. Biomed Environ Sci 28:751–764. doi:10.3967/bes2015.105 26582097

[4] Shah HJ, Jervis RH, Wymore K, Rissman T, LaClair B, Boyle MM, Smith K, Lathrop S, McGuire S, Trevejo R, McMillian M, Harris S, Zablotsky Kufel J, Houck K, Lau CE, Devine CJ, Boxrud D, Weller DL. 2024. Reported incidence of infections caused by pathogens transmitted commonly through food: impact of increased use of culture-independent diagnostic tests - Foodborne Diseases Active Surveillance Network, 1996-2023. MMWR Morb Mortal Wkly Rep 73:584–593. doi:10.15585/mmwr.mm7326a1 38959172 PMC11221634

[5] Bedenić B, Pospišil M, Nađ M, Bandić Pavlović D. 2025. Evolution of β-lactam antibiotic resistance in Proteus species: from extended-spectrum and plasmid-mediated AmpC β-lactamases to carbapenemases. Microorganisms 13:508. doi:10.3390/microorganisms13030508 40142401 PMC11946153

[6] Guo Y, Zou G, Kerdsin A, Schultsz C, Hu C, Bei W, Chen H, Li J, Zhou Y. 2024. Characterization of NMCR-3, NMCR-4 and NMCR-5, three novel non-mobile colistin resistance determinants: implications for MCR-3, MCR-7, and MCR-5 progenitors, respectively. Drug Resist Updat 75:101088. doi:10.1016/j.drup.2024.101088 38744111

[7] Galié S, García-Gutiérrez C, Miguélez EM, Villar CJ, Lombó F. 2018. Biofilms in the food industry: health aspects and control methods. Front Microbiol 9:898. doi:10.3389/fmicb.2018.00898 29867809 PMC5949339

[8] Sung K, Khan S, Ahn J. 2023. Foodborne pathogen biofilms: development, detection, control, and antimicrobial resistance. Pathogens 12:352. doi:10.3390/pathogens12020352 36839624 PMC9961813

[9] Abriat C, Enriquez K, Virgilio N, Cegelski L, Fuller GG, Daigle F, Heuzey MC. 2020. Mechanical and microstructural insights of Vibrio cholerae and Escherichia coli dual-species biofilm at the air-liquid interface. Colloids Surf B Biointerfaces 188:110786. doi:10.1016/j.colsurfb.2020.110786 31954270

[10] Elafify M, Liao X, Feng J, Ahn J, Ding T. 2024. Biofilm formation in food industries: challenges and control strategies for food safety. Food Res Int 190:114650. doi:10.1016/j.foodres.2024.114650 38945629

[11] Threlfall EJ. 2002. Antimicrobial drug resistance in Salmonella: problems and perspectives in food- and water-borne infections. FEMS Microbiol Rev 26:141–148. doi:10.1111/j.1574-6976.2002.tb00606.x 12069879

[12] Akshay SD, Deekshit VK, Mohan Raj J, Maiti B. 2023. Outer membrane proteins and efflux pumps mediated multi-drug resistance in Salmonella: rising threat to antimicrobial therapy. ACS Infect Dis 9:2072–2092. doi:10.1021/acsinfecdis.3c00408 37910638

[13] Ivanova M, Ovsepian A, Leekitcharoenphon P, Seyfarth AM, Mordhorst H, Otani S, Koeberl-Jelovcan S, Milanov M, Kompes G, Liapi M, et al.. 2024. Azithromycin resistance in Escherichia coli and Salmonella from food-producing animals and meat in Europe. J Antimicrob Chemother 79:1657–1667. doi:10.1093/jac/dkae161 38775752 PMC11215539

[14] Petrovic Fabijan A, Iredell J, Danis-Wlodarczyk K, Kebriaei R, Abedon ST. 2023. Translating phage therapy into the clinic: recent accomplishments but continuing challenges. PLoS Biol 21:e3002119. doi:10.1371/journal.pbio.3002119 37220114 PMC10204993

[15] Guo Y, Li J, Islam MS, Yan T, Zhou Y, Liang L, Connerton IF, Deng K, Li J. 2021. Application of a novel phage vB_SalS-LPSTLL for the biological control of Salmonella in foods. Food Res Int 147:110492. doi:10.1016/j.foodres.2021.110492 34399488

[16] Yan T, Liang L, Yin P, Zhou Y, Sharoba AM, Lu Q, Dong X, Liu K, Connerton IF, Li J. 2020. Application of a novel phage LPSEYT for biological control of Salmonella in foods. Microorganisms 8:400. doi:10.3390/microorganisms8030400 32178465 PMC7142823

[17] Huang C, Virk SM, Shi J, Zhou Y, Willias SP, Morsy MK, Abdelnabby HE, Liu J, Wang X, Li J. 2018. Isolation, characterization, and application of bacteriophage LPSE1 against Salmonella enterica in ready to eat (RTE) foods. Front Microbiol 9:1046. doi:10.3389/fmicb.2018.01046 29887839 PMC5982681

[18] Liu H, Gu W, Lu Y, Ding L, Guo Y, Zou G, Wu W, Zheng D, Liu C, Wang C, Cao Y, Li J. 2024. Exploration of phage–agrochemical interaction based on a novel potent phage LPRS20-targeting Ralstonia solanacearum. J Agric Food Chem 72:28005–28018. doi:10.1021/acs.jafc.4c03799 39360931

[19] Zhang Y, Zou G, Islam MS, Liu K, Xue S, Song Z, Ye Y, Zhou Y, Shi Y, Wei S, Zhou R, Chen H, Li J. 2023. Combine thermal processing with polyvalent phage LPEK22 to prevent the Escherichia coli and Salmonella enterica contamination in food. Food Res Int 165:112454. doi:10.1016/j.foodres.2022.112454 36869473

[20] Zou G, Ndayishimiye L, Xin L, Cai M, Zhang L, Li J, Song Z, Wu R, Zhou Y, Shi Y, Ye Y, Zhou R, Li J. 2023. Application of a novel phage LPCS28 for biological control of Cronobacter sakazakii in milk and reconstituted powdered infant formula. Food Res Int 172:113214. doi:10.1016/j.foodres.2023.113214 37689848

[21] Islam M, Yang X, Euler CW, Han X, Liu J, Hossen M, Zhou Y, Li J. 2021. Application of a novel phage ZPAH7 for controlling multidrug-resistant Aeromonas hydrophila on lettuce and reducing biofilms. Food Control 122:107785. doi:10.1016/j.foodcont.2020.107785

[22] Zhang X, Niu YD, Nan Y, Stanford K, Holley R, McAllister T, Narváez-Bravo C. 2019. SalmoFresh effectiveness in controlling Salmonella on romaine lettuce, mung bean sprouts and seeds. Int J Food Microbiol 305:108250. doi:10.1016/j.ijfoodmicro.2019.108250 31226567

[23] Shakeri G, Hammerl JA, Jamshidi A, Ghazvini K, Rohde M, Szabo I, Kehrenberg C, Plötz M, Kittler S. 2021. The lytic Siphophage vB_StyS-LmqsSP1 reduces the number of Salmonella enterica serovar typhimurium isolates on chicken skin. Appl Environ Microbiol 87:e0142421. doi:10.1128/AEM.01424-21 34586906 PMC8612259

[24] Bao H, Zhang H, Zhou Y, Zhu S, Pang M, Zhang X, Wang Y, Wang J, Olaniran A, Xiao Y, Schmidt S, Wang R. 2022. Dysbiosis and intestinal inflammation caused by Salmonella Typhimurium in mice can be alleviated by preadministration of a lytic phage. Microbiol Res 260:127020. doi:10.1016/j.micres.2022.127020 35462115

[25] Huang C, Shi J, Ma W, Li Z, Wang J, Li J, Wang X. 2018. Isolation, characterization, and application of a novel specific Salmonella bacteriophage in different food matrices. Food Res Int 111:631–641. doi:10.1016/j.foodres.2018.05.071 30007727

[26] Guenther S, Herzig O, Fieseler L, Klumpp J, Loessner MJ. 2012. Biocontrol of Salmonella Typhimurium in RTE foods with the virulent bacteriophage FO1-E2. Int J Food Microbiol 154:66–72. doi:10.1016/j.ijfoodmicro.2011.12.023 22244192

[27] Cao Y, Khanal D, Kim J, Chang RYK, Byun AS, Morales S, Banaszak Holl MM, Chan HK. 2023. Stability of bacteriophages in organic solvents for formulations. Int J Pharm 646:123505. doi:10.1016/j.ijpharm.2023.123505 37832702

[28] Webber B, Pottker ES, Rizzo NN, Núncio AS, Peixoto CS, Mistura E, Dos Santos LR, Rodrigues LB, do Nascimento VP. 2023. Surface conditioning with bacteriophages reduces biofilm formation of Salmonella Heidelberg. Food Sci Technol Int 29:275–283. doi:10.1177/10820132221074783 35075919

[29] Islam MS, Hu Y, Mizan MFR, Yan T, Nime I, Zhou Y, Li J. 2020. Characterization of Salmonella phage LPST153 that effectively targets most prevalent Salmonella serovars. Microorganisms 8:1089. doi:10.3390/microorganisms8071089 32708328 PMC7409278

[30] Chen X, Xi Y, Zhang H, Wang Z, Fan M, Liu Y, Wu W. 2016. Characterization and adsorption of Lactobacillus virulent phage P1. J Dairy Sci 99:6995–7001. doi:10.3168/jds.2016-11332 27372579

[31] Wang S, Mirmiran SD, Li X, Li X, Zhang F, Duan X, Gao D, Chen Y, Chen H, Qian P. 2023. Temperate phage influence virulence and biofilm-forming of Salmonella Typhimurium and enhance the ability to contaminate food product. Int J Food Microbiol 398:110223. doi:10.1016/j.ijfoodmicro.2023.110223 37120944

[32] Hosny RA, Shalaby AG, Nasef SA, Sorour HK. 2023. Antibiofilm activity of a lytic Salmonella phage on different Salmonella enterica serovars isolated from broiler farms. Int Microbiol 26:205–217. doi:10.1007/s10123-022-00294-1 36334144 PMC10148789

[33] Zeng X, Wang W, Zhu D, Liu M, Wang M, Jia R, Chen S, Yang Q, Wu Y, Zhang S, Huang J, Tian B, Ou X, Sun D, He Y, Wu Z, Cheng A, Zhao X. 2025. Two receptor-targeting mechanisms of lambda-like siphophage Gifsy-1 of Salmonella Typhimurium. PLoS Pathog 21:e1013352. doi:10.1371/journal.ppat.1013352 40743116 PMC12312913

[34] Gao D, Ji H, Li X, Ke X, Li X, Chen P, Qian P. 2023. Host receptor identification of a polyvalent lytic phage GSP044, and preliminary assessment of its efficacy in the clearance of Salmonella. Microbiol Res 273:127412. doi:10.1016/j.micres.2023.127412 37243984

[35] Yehl K, Lemire S, Yang AC, Ando H, Mimee M, Torres MDT, de la Fuente-Nunez C, Lu TK. 2019. Engineering phage host-range and suppressing bacterial resistance through phage tail fiber mutagenesis. Cell 179:459–469. doi:10.1016/j.cell.2019.09.015 31585083 PMC6924272

[36] Schmelcher M, Loessner MJ. 2016. Bacteriophage endolysins: applications for food safety. Curr Opin Biotechnol 37:76–87. doi:10.1016/j.copbio.2015.10.005 26707470

[37] Fischetti VA. 2018. Development of phage lysins as novel therapeutics: a historical perspective. Viruses 10:310. doi:10.3390/v10060310 29875339 PMC6024357

[38] Euler CW, Raz A, Hernandez A, Serrano A, Xu S, Andersson M, Zou G, Zhang Y, Fischetti VA, Li J. 2023. PlyKp104, a novel phage lysin for the treatment of Klebsiella pneumoniae, Pseudomonas aeruginosa, and other Gram-negative ESKAPE pathogens. Antimicrob Agents Chemother 67:e0151922. doi:10.1128/aac.01519-22 37098944 PMC10190635

[39] Zhang Y, Li R, Zou G, Guo Y, Wu R, Zhou Y, Chen H, Zhou R, Lavigne R, Bergen PJ, Li J, Li J. 2024. Discovery of antimicrobial lysins from the "dark matter" of uncharacterized phages using artificial intelligence. Adv Sci (Weinh) 11:e2404049. doi:10.1002/advs.202404049 38899839 PMC11348152

[40] Wu H, Chen R, Li X, Zhang Y, Zhang J, Yang Y, Wan J, Zhou Y, Chen H, Li J, Li R, Zou G. 2024. ESKtides: a comprehensive database and mining method for ESKAPE phage-derived antimicrobial peptides. Database (Oxford) 2024:baae022. doi:10.1093/database/baae022 38531599 PMC10965241

[41] Paudyal N, Anihouvi V, Hounhouigan J, Matsheka MI, Sekwati-Monang B, Amoa-Awua W, Atter A, Ackah NB, Mbugua S, Asagbra A, Abdelgadir W, Nakavuma J, Jakobsen M, Fang W. 2017. Prevalence of foodborne pathogens in food from selected African countries – A meta-analysis. Int J Food Microbiol 249:35–43. doi:10.1016/j.ijfoodmicro.2017.03.002 28271855

[42] Shen C, Islam MT, Masuda Y, Honjoh K-I, Miyamoto T. 2020. Transcriptional changes involved in inhibition of biofilm formation by ε-polylysine in Salmonella Typhimurium. Appl Microbiol Biotechnol 104:5427–5436. doi:10.1007/s00253-020-10575-2 32307570

[43] Zhao J, Lin Y, Wang C, Zayda M, Maung AT, Mohammadi TN, Duc HM, Yu P, Ma M, Gong D, Sato J, Masuda Y, Honjoh K-I, Miyamoto T, Zeng Z. 2023. Biocontrol of Salmonella Typhimurium in milk, lettuce, raw pork meat and ready-to-eat steamed-chicken breast by using a novel bacteriophage with broad host range. Int J Food Microbiol 402:110295. doi:10.1016/j.ijfoodmicro.2023.110295 37352774

[44] Yang Y, Li R, Zhong Q, Guo Y, Wu R, Chen H, Zhou R, Ye R, Dąbrowska K, Prajsnar TK, Stafford GP, Zou G, Zhou Y, Li J, Song Z. 2025. In situ gut microbiota editing: enhancing therapeutic efficacy for bacterial colitis by compatible oral hydrogel microspheres with phages. Nat Commun 16:9785. doi:10.1038/s41467-025-65498-1 41198711 PMC12808749

[45] Byun KH, Han SH, Choi MW, Kim BH, Park SH, Ha SD. 2022. Biofilm eradication ability of phage cocktail against Listeria monocytogenes biofilms formed on food contact materials and effect on virulence-related genes and biofilm structure. Food Res Int 157:111367. doi:10.1016/j.foodres.2022.111367 35761627

[46] Ivers C, Yucel U, Boyle D, Trinetta V. 2024. Evaluation of Salmonella biofilm attachment and hydrophobicity characteristics on food contact surfaces. BMC Microbiol 24:387. doi:10.1186/s12866-024-03556-2 39363349 PMC11447956

