# Supplementary material for: Antibiofilm efficacy of phage W5 against antimicrobial-resistant Salmonella Typhimurium targeting biofilms in dairy/meat/egg and on food-processing surfaces (PP/PE)
Source: Appl Environ Microbiol. 2026 Mar 26;92(4):e01878-25. doi: 10.1128/aem.01878-25 (PMC13101489; doi:10.1128/aem.01878-25)
Supplement: Table S1 — Antibiotic resistance profiles of S. Typhimurium CMCC 50115. [file aem.01878-25-s0001.docx]

Supplementary materials

**Antibiofilm Efficacy of phage W5 Against Antimicrobial-Resistant Salmonella Typhimurium Targeting Biofilms in Dairy/Meat/Egg and on Food-Processing Surfaces (PP/PE)**

Qian Chong, Qing Cao, Xi Wang, Ziqiu Fan, Yonghui Ma, Kunzhong Zhang, Jing Deng, Xuehui Zhao, Ji Zhi, Haohao Zhang, Kaihui Yang, Huiwen Xue, Huitian Gou*.

College of Veterinary Medicine, Gansu Agricultural University, LanZhou 730000, Gansu,China

**Supplementary Table S1:Antibiotic Resistance Profiles of S. Typhimurium CMCC 50115**

| Type | | Antibiotic | Resistant (R) | Intermediate (I) | Susceptible (S) | Inhibition Zone Diameter (mm)  CMCC 50115 CMCC 50071 |  |
| --- | --- | --- | --- | --- | --- | --- | --- |
| **Beta-lactams** | **Penicillins** | Ampicillin | ≤13 | 14~16 | ≥17 | 22 | |
|  | **Cephalosporins** | Cefazolin | ≤14 | 15~17 | ≥18 | 20 | |
|  |  | Cephalexin | ≤14 | 15~17 | ≥18 | 18 | |
|  |  | Ceftriaxone | ≤13 | 14~20 | ≥21 | 26 | |
| **Aminoglycosides** | | Streptomycin | ≤11 | 12~14 | ≥15 | 10 | |
|  |  | Kanamycin | ≤13 | 14~17 | ≥18 | 18 | |
|  |  | Gentamicin | ≤12 | 13~14 | ≥15 | 18 | |
|  |  | Amikacin | ≤14 | 15~16 | ≥17 | 20 | |
| **Polymyxins** | | Polymyxin B | ≤10 | 11~15 | ≥16 | 15 | |
| **Macrolides** | | Erythromycin | ≤12 | 13~14 | ≥15 | 0 | |
| **Lincosamides** | | Lincomycin | ≤12 | 13~20 | ≥21 | 0 | |
| **Glycopeptides** | | Vancomycin | ≤12 | 13~14 | ≥15 | 0 | |
| **Quinolones** | | Ciprofloxacin | ≤15 | 16~20 | ≥21 | 24 | |
|  |  | Ofloxacin | ≤14 | 15~19 | ≥20 | 23 | |
| **Tetracyclines** | | Tetracycline | ≤14 | 15~18 | ≥19 | 15 | |
|  |  | Minocycline | ≤14 | 15~18 | ≥19 | 14 | |
